# Tetraacylstannanes as Long‐Wavelength Visible‐Light Photoinitiators with Intriguing Low Toxicity

**DOI:** 10.1002/chem.201801622

**Published:** 2018-05-29

**Authors:** Judith Radebner, Anna Eibel, Mario Leypold, Nina Jungwirth, Thomas Pickl, Ana Torvisco, Roland Fischer, Urs Karl Fischer, Norbert Moszner, Georg Gescheidt, Harald Stueger, Michael Haas

**Affiliations:** ^1^ Institute of Inorganic Chemistry Technische Universität Graz Stremayrgasse 9/IV 8010 Graz Austria; ^2^ Institute of Physical and Theoretical Chemistry Technische Universität Graz Stremayrgasse 9/IV 8010 Graz Austria; ^3^ Ivoclar Vivadent AG Bendererstrasse 2 9494 Schaan Liechtenstein

**Keywords:** acylstannanes, organostannanes, photochemistry, photoinitiators, radical polymerization

## Abstract

The first tetraacylstannanes Sn[(CO)R]_4_ (*R*=2,4,6‐trimethylphenyl (**1 a**) and 2,6‐dimethylphenyl (**1 b**)), a class of highly efficient Sn‐based photoinitiators, were synthesized. The formation of these derivatives was confirmed by NMR spectroscopy, mass spectrometry, and X‐ray crystallography. The UV/Vis absorption spectra of **1 a**, **b** reveal a significant redshift of the longest wavelength absorption compared to the corresponding germanium compounds. In contrast to the known toxicity of organotin derivatives, the AMES test and cytotoxicity studies reveal intriguing low toxicity. The excellent performance of **1** as photoinitiators is demonstrated by photobleaching (UV/Vis) and NMR/CIDNP investigations, as well as photo‐DSC studies.

From the Bronze Age (3500 BC) onward, tin has been a highly appreciated metal and raw material for further processing. Its acquisition marks an important part in the evolution of mankind. Pioneering works in organometallic tin chemistry by Frankland,^1^ Cadet,^2^ or Zeise^3^ sparked the interest into organic tin derivatives. Advances in analytical techniques, including Sn NMR, X‐ray diffraction, or appropriate computational methods aided organotin chemistry to be a thriving field of research. Besides being used as fungicides, insecticides or stabilizers in polyvinyl chloride (PVC), organostannanes are widely used in chemical synthesis. However, due to the acute toxicity of organotin compounds, their use in synthetic chemistry has been connoted quite negatively and gradually diminished related research activities. Hitherto, the interest in acylstannane chemistry remained modest because of a lack of suitable synthetic methods towards such. For a long time, acylstannanes were virtually unknown and believed to be extremely labile. For the sake of completeness, these derivatives were mentioned as a side note when reporting on acylsilanes and acylgermanes.^4^ Recent years have witnessed intense scientific efforts in the chemistry of keto‐derivatives of main group IV organometalloids (mainly, Ge based) and their application as visible light (VL) photoinitiators.^5^, ^6^, ^7^, ^8^ The search for new photoinitiators (PIs) for radical polymerization exhibiting enhanced reactivity remains of great interest.^9^ To date, phosphorous‐based initiators (mono‐ and bisacylphosphane oxides) are well established, although UV light is required for sufficient photocuring and the parent compounds, as well as their photoproducts are in most cases highly toxic.^10^, ^11^, ^12^ Thus, germanium‐centered PI systems have emerged over the past few years as promising alternatives due to their low toxicity and the bathochromic shift of their longest‐wavelength absorption bands.^6^, ^13^, ^14^, ^15^ However, a significant drawback of the latter is the low abundance of germanium in the earth crust, resulting in high prices of germanium‐based PIs. Therefore, this system cannot fully meet the requirements for photoinitiators in high‐throughput polymer synthesis.

To overcome the above‐mentioned restrictions, we investigated the implementation of other central atoms. The replacement of germanium by silicon leads to the formation of tetra‐acylsilanes. But these derivatives appeared to be ineffective for free‐radical photopolymerization.^16^, ^17^ Herein, we pioneer the use of tin as the central atom in metallo‐acyl‐based photoinitiators, tremendously extending the scope of previously established systems in terms of long‐wavelength absorption in combination with cost‐effective synthesis and excellent biocompatibility.

We report the outstanding potential of tetraacylstannanes **1 a**, **b** as a unique class of long‐wavelength visible‐light PIs for free‐radical polymerization. Tetraacylstannanes **1 a**, **b** are accessible in a remarkably easy‐to‐perform one‐pot reaction (Scheme 1). Notably, this new PI system shows very low cytotoxicity (XTT_50_ value of 108.53 μg mL^−1^), in line with its low water solubility.^18^ Moreover, the bacterial reverse‐mutation test, the so‐called Ames test, proved that **1** does not induce gene mutations, which is remarkable for organotin compounds.^19^ Structurally related acylgermanes show similar low cytotoxicity (e.g., benzoyltrimethylgermane: XTT_50_ 215 μg mL^−1^).^8^, ^14^ In contrast, for the widely applied phosphorus‐based PIs (e.g., Irgacure 819) barely any reports on cytotox and AMES tests are available.^12^, ^20^ However, a similar acylphosphine oxide^21^ was reported to show a higher cytotox (XTT_50_ 69.1 μg mL^−1^)^22^ than Sn or Ge compounds. Additionally, photoproducts of P‐based PIs are known to be highly toxic.^23^ In this context, acylgermanes and acylstannanes are promising PIs for applications requiring biocompatibility.

**Scheme 1:**
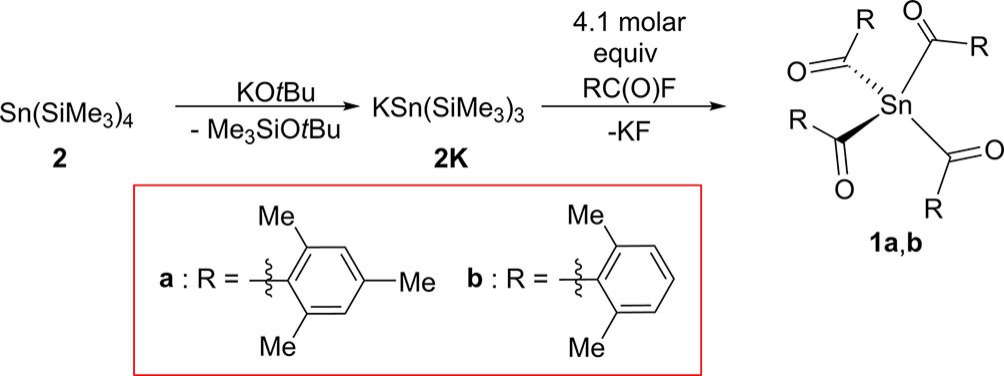
Synthesis of tetraacylstannanes **1 a** and **b**.

Recently, our group published a series of papers on previously unknown acylgermanes.^6^, ^24^, ^25^, ^26^ Exploiting the multiple silyl abstraction enabled the targeted synthesis of these highly efficient PIs in high yields. Moreover, the strength of this reaction sequence lies in its high functional‐group tolerance.^24^, ^25^

Following the results obtained for tetraacylgermanes, we discovered that the reaction of potassium stannide **2K** 27 with 4.1 molar equivalent of acid fluorides F−(CO)R (R=aryl) leads to the formation of tetraacylstannanes **1**. Mechanistically speaking, **2K** undergoes a salt metathesis reaction with the respective acid fluoride, in which KF and tris(trimethylsilyl)acylstannane **3** are formed.

The consecutive nucleophilic attack of the fluoride ion on **4**, originating from the formation of **2K**, in situ generates Me_3_SiF **5** and *t*BuO^−^, which further reacts with **3** to form the stannenolate **6**. Accordingly, **4** and **5** could be detected by NMR spectroscopy in the reaction mixture (*δ* ^29^Si=30.3 and 6.92 ppm, respectively).^28^ Subsequently, the applied excess of acid fluoride immediately reacts with **6** to the respective bisacylstannane **7** releasing again KF and **4**. Acylation proceeds until all trimethylsilyl groups are abstracted, and the final product **1** is formed (Scheme 2). It appears that di‐*ortho* substitution at the phenyl ring is necessary for the successful preparation of tetraacylstannanes. To date, no derivatives with a different substitution pattern were isolated. We assume that the di‐*ortho* substitution prevents the nucleophilic attack of any reactive anionic intermediate formed (F^−^ or *t*BuO^−^). A characteristic feature of tetraacylstannanes **1** is the ^13^C NMR resonance for the carbonyl group, which appears between 243 and 244 ppm. A similar tendency was found for tetraacylgermanes and ‐silanes.^6^, ^16^

**Scheme 2:**
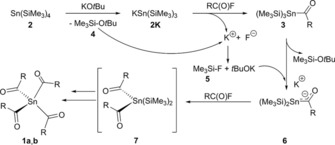
Proposed mechanism for the synthesis of tetraacylstannanes.

The analytical data for **1 a** and **b** (see the Supporting Information) are consistent with the proposed structures. As a representative example, the structure of **1 a** is depicted in Figure 1. The structural features of these tin derivatives correlate with the results obtained for acylgermanes. Di‐*ortho* substitution induces a significant torsion between the carbonyl group and the aromatic ring plane. The Sn−C bond length is similar to the values found for other Sn−C single bonds.^29^

**Figure 1:**
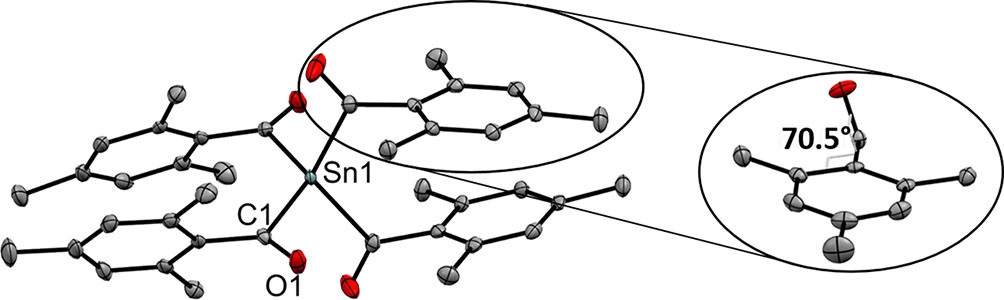
ORTEP representation of **1 a**. Thermal ellipsoids are drawn at the 50 % probability level. Hydrogen atoms are omitted for clarity. Selected bond lengths [Å] and angles [°] with estimated standard deviations: Sn(1)−C(1) 2.244(2), Sn(1)−C(11) 2.247(2), Sn(1)−C(21) 2.247(2), Sn(1)−C(31) 2.256(2), C−O (mean) 1.208; C‐Ge‐C (mean) 111.7; O‐C‐C_aryl_‐C_aryl_ (mean) 70.5.

As was mentioned above, photopolymerization of biocompatible materials requires non‐toxic PIs and non‐toxic irradiation sources (visible light). In the course of our studies, we found that the absorption maximum of non‐toxic tetraacylgermanes show values between 363 and 422 nm.^24^ However, tetraacylgermanes lack sufficient curing efficiency upon irradiation with light sources operating above 450 nm. Nevertheless, this type I PI system represents the most bathochromic shifted absorption bands to date. This border is shattered by the introduction of tetraacylstannanes **1**. Figure 2 illustrates the longest‐wavelength absorption bands, which were computationally assigned to the HOMO–LUMO transition and show considerable charge‐transfer character. Upon excitation, electron density is displaced from the n(C=O)/σ(Sn−C) bonding HOMO to the π*(C=O/Aryl) antibonding LUMO (Figure 3), which results in the population of an orbital with antibonding character between the Sn−C bond.

**Figure 2:**
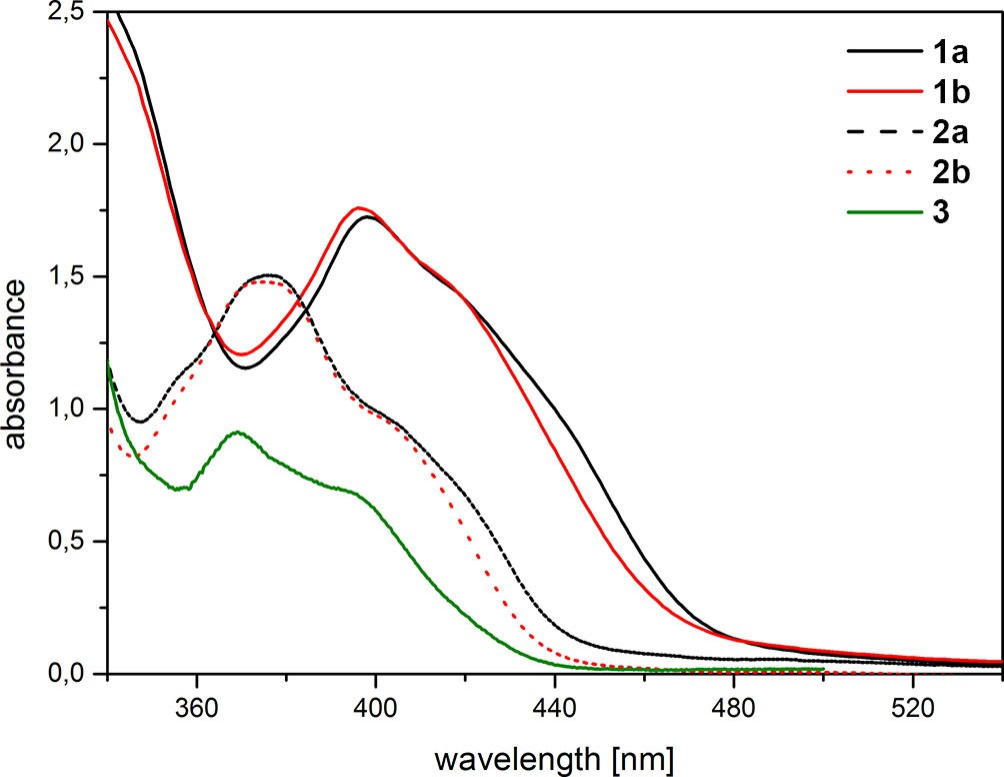
UV/Vis absorption spectra of **1 a**, **b** and **2 a**, **b** (the respective germanium‐analogues) and **3** (bisacylphosphane oxide Irgacure 819; chloroform solution, *c*=10^−3^ mol L^−1^).

**Figure 3:**
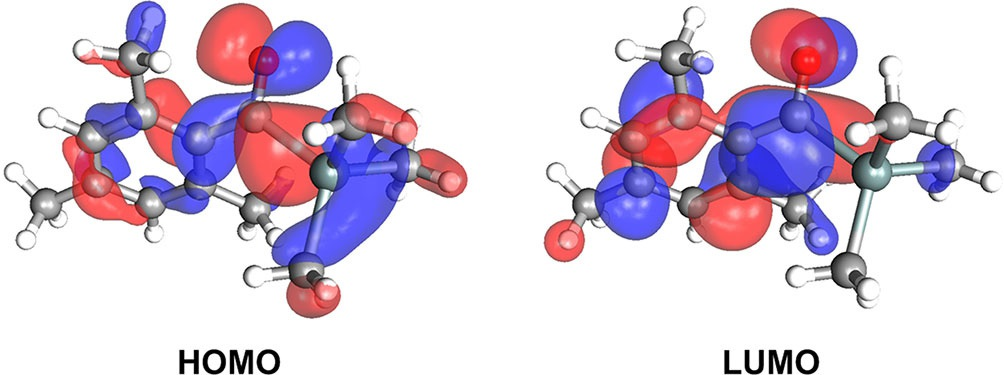
HOMO (left) and LUMO (right) of mesitoyltrimethylstannane, a simplified version of **1 a**, to illustrate the Frontier orbitals of tetraacylstannanes **1 a** and **b**.

In comparison to the respective germanium analogues **2 a** and **b** and to bisacylphosphine oxide **3** (Scheme 3), the extinction coefficient of **1 a** and **b** are significantly increased. The bathochromic shifted *λ*_max_, as well as the increased extinction coefficient, induce a more pronounced tailing into the visible‐light region >450 nm, enabling α‐cleavage upon irradiation with light sources operating >450 nm. Hence, **1** represents a radical source with most promising absorption properties for visible‐light applications (Table 1).

**Scheme 3:**
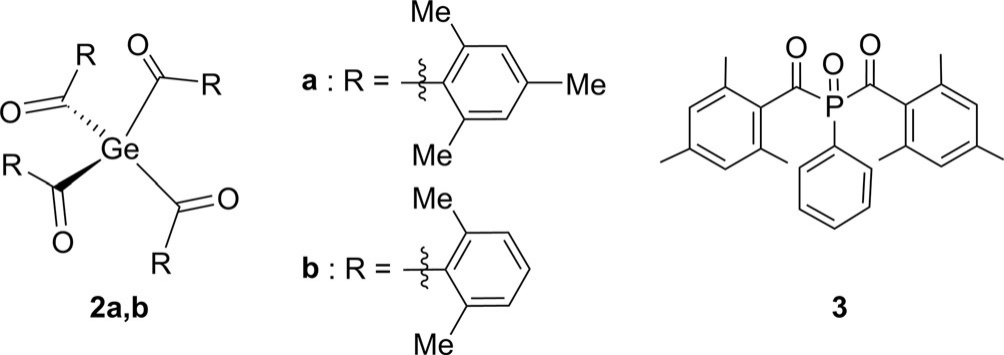
Reference Compounds: Tetraacylgermanes **2 a**,**b** and bisacylphosphine oxide **3**.

**Table 1:** Experimental and PCM(CHCl_3_) TD‐DFT CAM‐B3LYP/LANL2DZdp‐ECP(Sn),def2‐TZVP(H,C,O)//B3LYP/LANL2DZdp‐ECP(Sn),6‐31+G(d)(H,C,O) calculated wavelength absorption maxima, *λ* [nm], extinction coefficients *ϵ* [dm^2^ mol^−1^], and oscillator strengths *f* for **1 a** and **b** (CHCl_3_).

	*λ*_max, exptl_ (*ϵ*)	*λ*_max, calcd_ (*f*)	Absorption edge	Assignment
**1 a**	398 (1776)	416, 409, 389 (0.0221, 0.0051, 0.0137)	480	n/σ‐π*(CO/Aryl)
**1 b**	396 (1735)	417, 409, 389 (0.0167, 0.0053, 0.0131)	480	n/σ‐π*(CO/Aryl)

To assess the efficiency of **1 a** and **b** as radical photoinitiators, we have performed photobleaching experiments.^6^, ^7^ Steady‐state photolysis (SSP) upon irradiation with an LED operated at 470 nm reveals remarkably fast bleaching of **1 a** and **b** compared to germanium‐5, 6, 7, 15 and phosphorus‐based^10^, ^30^ photoinitiators **2** and **3** (Figure 4). Fast photobleaching of the initiator is an indication for efficient radical formation and is moreover crucial for achieving high curing depths and colorless polymers.^31^ Compound **1 b** exhibits the fastest photobleaching, whereas bisacylphosphane oxide **3** shows almost no bleaching, being unsuitable for curing applications in this wavelength region. The superior photobleaching performance of tetraacylstannanes **1** over the reference initiators **2** and **3** upon irradiation with high wavelength visible light (470 nm) is consistent with the bathochromically shifted absorption spectra (Figure 2).

**Figure 4:**
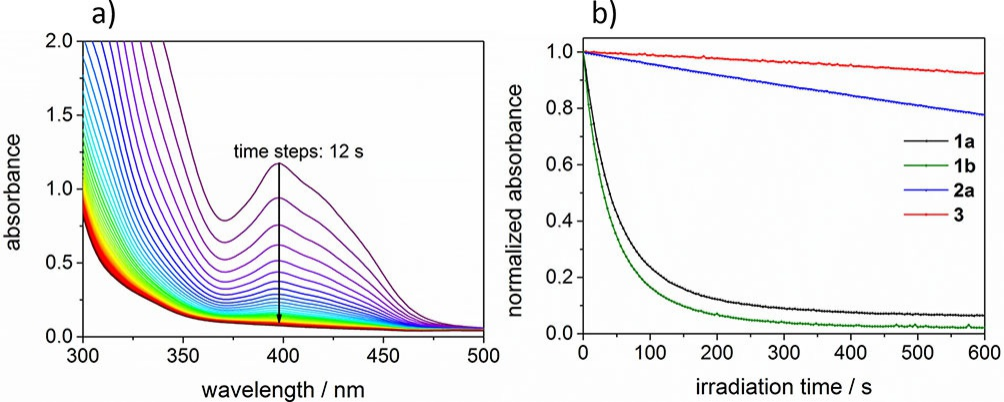
a) Photobleaching of **1 a** upon irradiation at 470 nm. b) Plots of normalized absorbance versus time for compounds **1 a**, **b**, **2 a**, and **3**, monitored at absorption maxima (**1 a** 398 nm; **1 b** 396.5 nm; **2 a** 375 nm; **3** 369.5 nm; **3** 409 nm). Samples: 0.6 mm PI in acetonitrile.

We have investigated the radical reaction pathways upon irradiation of **1 a** and **b** in presence of monomers by using chemically induced dynamic nuclear polarization (CIDNP) NMR spectroscopy. This method allows following the α‐cleavage of the photoinitiators **1 a** and **b** and provides information about reaction products formed through radical pairs (**Sn** ^.^/**B** ^.^; Scheme 4). Radical‐pair‐based phenomena lead to enhanced absorptive or emissive NMR signals of reaction products, caused by a non‐Boltzmann population of magnetic energy levels.^5^, ^23^ Figure 5 compares the ^1^H NMR and CIDNP spectra of **1 a** recorded in presence of butyl acrylate **BA** (see the Supporting Information for the corresponding spectra of **1 b**). Polarized signals of the hydrogen atoms of the parent compound (at *δ*=2.25 ppm (1), *δ*=6.67 ppm (2), and *δ*=1.99 ppm (3)) can be attributed to the (cage) re‐formation of **1 a**, indicating a partly reversible α‐cleavage. The characteristic singlet at *δ*=10.5 ppm appearing in enhanced absorption corresponds to the aldehyde proton of the benzaldehyde derivative **BH**, clearly indicating α‐cleavage of **1 a** (Scheme 4 and Figure 5). Analogous signals have been observed in the CIDNP spectra of several photoinitiating systems containing a benzoyl moiety.^32^ The formation of **BH** is attributed to a disproportionation reaction (β‐hydrogen transfer) between a benzoyl‐type radical and radicals, which are able to donate hydrogen atoms (growing polymer chain).^32^, ^33^ Figure 5 additionally shows a strongly polarized emissive multiplet at *δ*=4.49 ppm and an absorptive multiplet at *δ*=3.37 ppm. These signals are tentatively assigned to the methylene protons of species **SnBAB**, which is formed upon addition of benzoyl radical **B^.^** to a chain radical initiated by **Sn** ^.^ (Figure 5). The analogous photoproduct has been observed for tetra‐acylgermanes.^6^ We assume that rotation around the C−C bond derived from **BA** (marked in pink in the structure of **SnBAB**, Figure 5) is sterically hindered by the adjacent bulky mesitoyl group, making the signal at *δ*=4.49 ppm appear as a doublet of doublets, instead of a triplet.

**Scheme 4:**
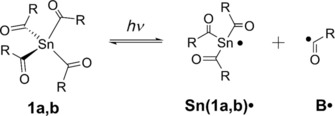
α‐Cleavage of tetraacylstannane derivatives **1 a** and **b**, leading to the formation of a tin‐centered radical **Sn** ^.^ and a benzoyl‐type radical **B** ^.^.

**Figure 5:**
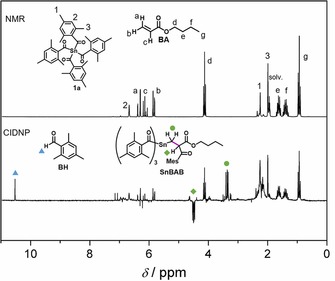
^1^H NMR and CIDNP spectra of **1 a** (10 mm solution in [D_3_]acetonitrile), recorded in the presence of butyl acrylate monomer (**BA**, 50 mm).

The prepared compounds were also investigated by means of photo‐DSC measurements to get fast and accurate information on their initiation efficiency. With a single photo‐DSC measurement, various significant parameters are accessible. From the height of the exothermic peak, the rate of polymerization *R*_P_ (mol L^−1^ s^−1^) can be calculated. The overall heat evolved (Δ*H*_P_) gives information on the final double conversion (DBC). Furthermore, the time to reach the maximum heat flow (*t*_max_) can be derived from the photo‐DSC plots. Herein, the photocuring of the crosslinking monomer 1,6‐hexandiol diacrylate (HDDA) with **1 a** was investigated (**1 a**: *t*_max_≈4.5 s, *R*_P,max_>0.31 mol L^−1^ s^−1^, DBC≈59 %). The previously reported tetraacylgermane **2 a** was used as reference compound, showing comparable reactivity to **1 a** under the chosen experimental conditions (**2 a**: *t*_max_≈5.1s, *R*_P,max_>0.25 mol L^−1^ s^−1^, DBC≈57 %). Photo‐DSC and conversion plots are depicted in Figure 6. A summary of the DSC parameters can be found in the Supporting Information.

**Figure 6:**
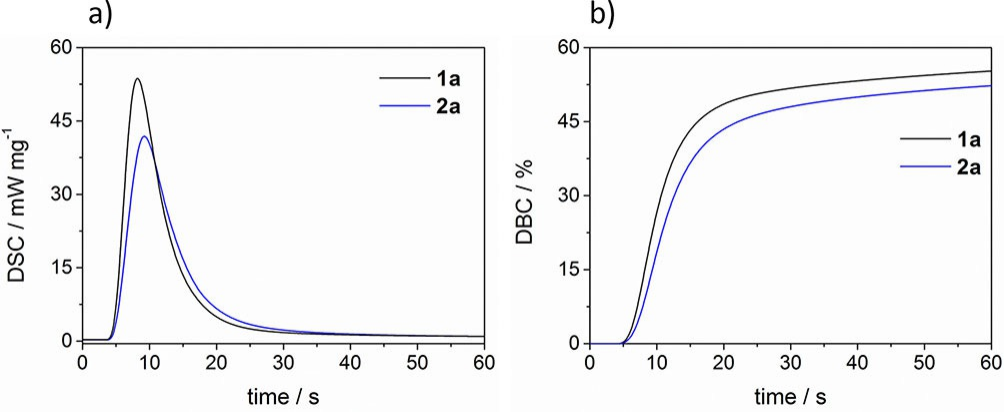
Photo‐DSC (a) and conversion plots (b) for the photopolymerization of HDDA with 0.3 w % photoinitiator. Samples were stabilized with 1000 ppm BHT prior to the measurement to prevent spontaneous polymerization.

In summary, we could synthesize and fully characterize the first examples of tetraacylstannanes, representing a class of highly efficient VL PIs. Unprecedentedly, the found photochemical properties are beneficial over those known for Ge‐ or P‐based PIs. Additionally, the low toxicity gives rise to any application, in which biocompatibility and costs are an issue. SSP experiments show that the tetraacylstannanes **1 a** and **b** are promising initiators for high‐wavelength visible‐light curing. CIDNP experiments confirm α‐cleavage of **1 a** and **b** and efficient addition of tin‐centered radicals and benzoyl radicals to monomer double bonds. High activity as photoinitiator was further verified by photo‐DSC experiments.

## Conflict of interest

The authors declare no conflict of interest.

## Supporting information

As a service to our authors and readers, this journal provides supporting information supplied by the authors. Such materials are peer reviewed and may be re‐organized for online delivery, but are not copy‐edited or typeset. Technical support issues arising from supporting information (other than missing files) should be addressed to the authors.

Supplementary
