# Supplementary material for: Tetraacylstannanes as Long‐Wavelength Visible‐Light Photoinitiators with Intriguing Low Toxicity
Source: Chemistry. 2018 May 29;24(33):8281–5. doi: 10.1002/chem.201801622 (PMC6032833; doi:10.1002/chem.201801622)
Supplement: Supplementary file 1 — Supplementary [file CHEM-24-8281-s001.pdf]

CHEMISTRY
A European Journal

Supporting Information

Tetraacylstannanes as Long-Wavelength Visible-Light Photoinitiators with Intriguing Low Toxicity

Judith Radebner,*[a] Anna Eibel,[b] Mario Leypold,[a] Nina Jungwirth,[a] Thomas Pickl,[a] Ana Torvisco,[a] Roland Fischer,[a] Urs Karl Fischer,[c] Norbert Moszner,[c] Georg Gescheidt,[b] Harald Stueger,[a] and Michael Haas*[a]

chem_201801622_sm_miscellaneous_information.pdf

CONTENT

Experimental Section 2
General Considerations 2
Synthesis of **1a** 3
Synthesis of **1b** 3
Analytical Section 4
NMR-Spectroscopy 4
Steady-State Photolysis (SSP) / UV-Vis Spectroscopy 6
CIDNP Spectroscopy 6
X-ray Crystallography 7
Photo-DSC 8
Computational Section 8
References 31

Experimental Section

General Considerations

All experiments were performed under a nitrogen atmosphere using standard Schlenk techniques. Solvents were dried using a column solvent purification system.[1] Commercial KOtBu (97%), 2,4,6-Trimethylbenzoyl chloride (99%) and 2,6-Dimethylbenzoic acid (97%) were used without any further purification.
^{1}H (299.95 MHz), ^{13}C (75.43 MHz) and ^{29}Si (59.59 MHz) NMR spectra were recorded on a Varian INOVA 300 spectrometer in C_6D_6 or $CDCl_3$ solution and referenced versus TMS using the internal ^{2}H-lock signal of the solvent. Tetrakis(trimethylsilyl)stannane, 2,4,6-Trimethylbenzoyl fluoride and 2,6-Dimethylbenzoyl fluoride were prepared according to published procedures.[2]

HRMS spectra were run on a Kratos Profile mass spectrometer. Infrared spectra were obtained on a Bruker Alpha-P Diamond ATR Spectrometer from the solid sample. Melting points were determined using Stuart SMP50 apparatus and are uncorrected. Elemental analyses were carried out on a Hanau Vario Elementar EL apparatus.

CIDNP experiments were carried out on a 200 MHz Bruker AVANCE DPX spectrometer equipped with a custom-made CIDNP probe head. A Hg-Xe UV lamp (Hamamatsu Lightningcure LC4, 3500 mW/cm^2, λ_{max} = 365 nm) served as the light source. The typical CIDNP timing sequence consists of the following parts: composite pulse presaturation, lamp flash, 90° radiofrequency detection pulse (2.2 µs), and FID. "Dummy" CIDNP spectra employing the same pulse sequence but without the lamp pulse were always measured and subtracted from the CIDNP spectra. Samples were prepared in deuterated acetonitrile and deoxygenated by bubbling with argon before the experiment. Chemical shifts (δ) are reported in ppm relative to tetramethylsilane (TMS) using the residual deuterated solvent signals as an internal reference (CD_3CN, δ_H = 1.94 ppm).

UV absorption spectra were recorded on a Perkin Elmer Lambda 5 spectrometer. Photobleaching experiments (steady state photolysis, SSP) were performed on a TIDAS UV- VIS spectrometer (J&M, Germany). Samples (0.6 mM photoinitiator in acetonitrile) were irradiated in a quartz cuvette (1 cm x 1 cm) from the side with a 470 nm LED (Roithner Lasertechnik, Vienna) while UV-Vis spectra were recorded. A spectroradiometer (GL Spectis, GL Optics, Germany) was used to measure the output power of the LED and to ensure an equal photon flux for SSP experiments (13.1 mW, photon flux: 0.05 µmol s^{-1} at 20 mA, peak wavelength: 462.5 nm).

Materials and Solvents for Spectroscopic Investigations. Bis(acyl)phosphane oxide BAPO Irgacure 819 (**3**) was obtained from TCI. Tetraacylgermanes **2a** and **2b** were synthesized as described previously.[3] The monomer butyl acrylate (BA, purity ≥ 99.0 %) was obtained from Fluka and freed from stabilizers by passing through a column of basic alumina before use. The solvents acetonitrile (MeCN, Sigma Aldrich, ≥ 99.9 %), and CD_2Cl_2 (Sigma Aldrich, 99.9 %) were employed as received. All experiments were performed at ambient temperature.

Photo-DSC. Experiments were performed on a photo-DSC instrument from Perkin Elmer (DSC 7). All formulations were analyzed in triplicate. After an equilibration phase

(4 min) the samples were irradiated for 3 min with blue LED light (Bluephase Lamp, 1200 mW·cm^{-2}, Ivoclar Vivadent AG) at 30°C under constant N_2 flow (20 mL min^{-1}). The heat flow of the polymerization reaction was recorded as a function of time. From the theoretical heat of polymerization of the monomer HDDA ($\Delta H_{0,HDDA}$ = 761.92 J g^{-1} or 172.4 kJ mol^{-1} determined from 86.2 kJ mol^{-1} per acrylate unit)[4]. The double bond conversion (DBC) was calculated by dividing the measured heat of polymerization ΔH through $\Delta H_{0,HDDA}$. The maximum rate of polymerization $R_{P,max}$ was determined using equation 1, with h as the height of the exothermic polymerization signal in mW mg^{-1} and ρ = 1010 g L^{-1} as the density for HDDA at 25 °C.[5]

$$R_{P,max} = \frac{h \times \rho}{\Delta H_{0,HDDA}} \quad (1)$$

Synthesis of 1a

A solution of $(Me_3Si)_3SnK$ in 10 mL of DME was freshly prepared from 1.00 g $Sn(SiMe_3)_4$ (2.43 mmol) and 0.30 g KOtBu (2.67 mmol) and slowly added to a solution of 1.66 g (9.96 mmol) 2,4,6-trimethylbenzoyl fluoride in 15 mL DME at 0°C. The mixture was stirred for another 10 min. For the work up the volatile components were evaporated under reduced pressure. The solid residue was extracted with pentane/toluene and filtered. The filtrate was pumped down. The obtained crude product was recrystallized from toluene, giving orange crystals of **1a**. Yield: 0.86 g (50%) of analytically pure **1a** as orange crystals.

mp: 122-125°C. **Anal. Calc.**: C, 67.91; H, 6.27 % **Found**: C, 67.24; H, 5.99 %. **^{119}Sn-NMR** ($CDCl_3$, TMS, ppm): -499.02. **^{13}C-NMR** ($CDCl_3$, TMS, ppm): 243.88 (C=O); 143.71, 139.53, 131.95, 128.99 (Mes-C); 21.33, 18.84 (Mes-CH_3).**^{1}H-NMR** ($CDCl_3$, TMS, ppm): 6.61 (s, 2H, Mes-H); 2.24 (s, 3H, Mes-CH_3); 2.05 (s, 6H, Mes-CH_3). IR (neat): v(C=O) = 1662, 1622 cm^{-1}. **HRMS**: **Calc**. 561.1458 for (M^+-Acyl) **Found**: 561.1450. **UV-VIS**: λ [nm] (ε [L mol^{-1} cm^{-1}]) = 399 (1727).

Synthesis of 1b

A solution of $(Me_3Si)_3SnK$ in 10 mL of DME was freshly prepared from 1.00 g $Sn(SiMe_3)_4$ (2.43 mmol) and 0.30 g KOtBu (2.67 mmol) and slowly added to a solution of 1.52 g (9.96 mmol) 2,6-dimethylbenzoyl fluoride in 15 mL DME at 0°C. The mixture was stirred for another 10 min. For the work up the volatile components were evaporated under reduced pressure. The solid residue was extracted with pentane/toluene and filtered. The filtrate was pumped down. The obtained crude product was recrystallized from toluene, giving orange crystals of **1b**. Yield: 0.98 g (62%) of analytically pure **1b** as orange crystals.

mp: 130-135°C. **Anal. Calc.**: C, 66.38; H, 5.57 % **Found**: C, 66.03; H, 5.81 %. **^{119}Sn-NMR** (C_6D_6, TMS, ppm): -505.88. **^{13}C-NMR** (C_6D_6, TMS, ppm): 243.18 (C=O); 146.99,

131.88, 129.66, 128.66 (Aryl-C); 18.82 (Aryl-*o*CH_3).**^{1}H-NMR** (C_6D_6, TMS, ppm): 7.05 (t, 1H, Aryl-H); 6.84 (s, 2H, Aryl-H); 2.04 (s, 6H, Aryl-*o*CH_3). IR (neat): ν(C=O) = 1669, 1633 cm^{-1}. **HRMS**: **Calc**. 519.1027 for (M^+-Acyl) **Found**: 519.0988 **UV-VIS**: λ [nm] (ε [L mol^{-1} cm^{-1}]) = 396 (1758).

Analytical Section

NMR-Spectroscopy

Figure S1: ^{1}H-, ^{13}C- and ^{119}Sn-NMR spectra of **1a** ($CDCl_3$ solution, vs. ext. TMS, ppm)

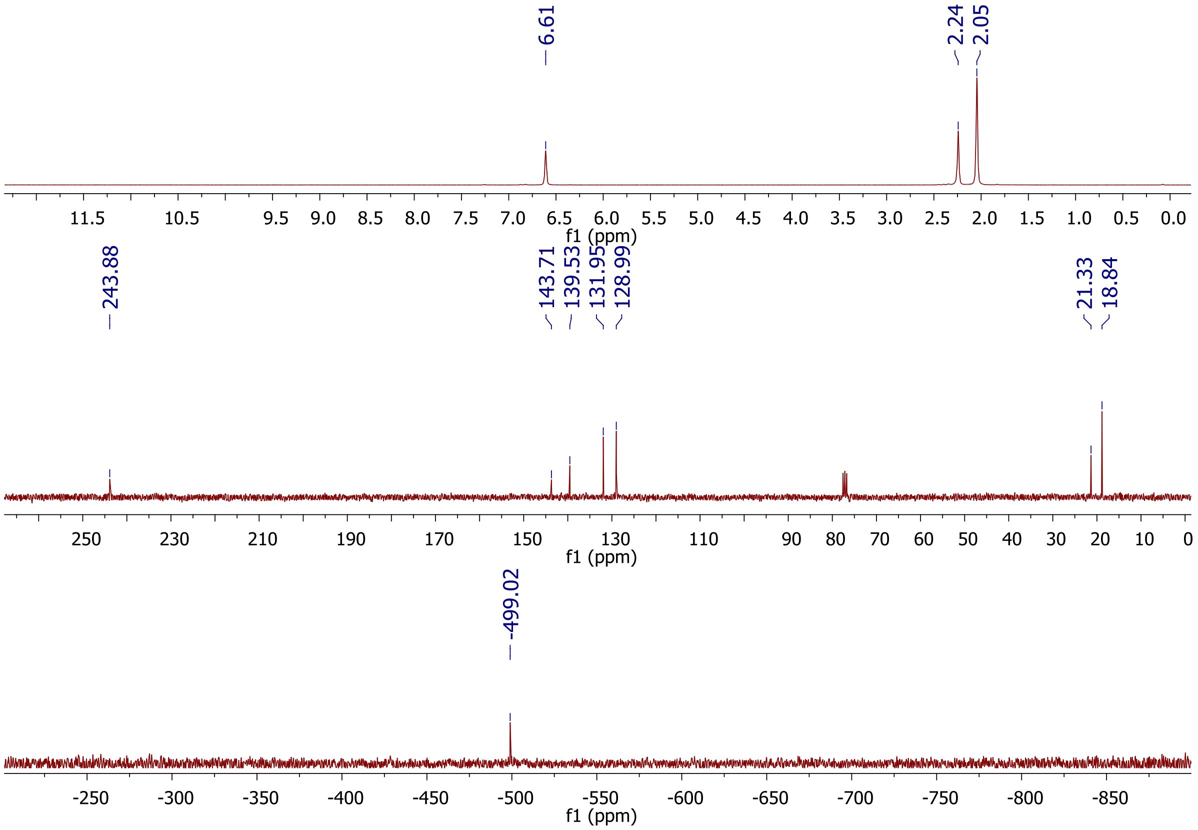

Figure S2: ^{1}H-, ^{13}C-, ^{119}Sn and 2D-HSQC-NMR spectra of **1b** (C_6D_6 solution, vs. ext. TMS, ppm)

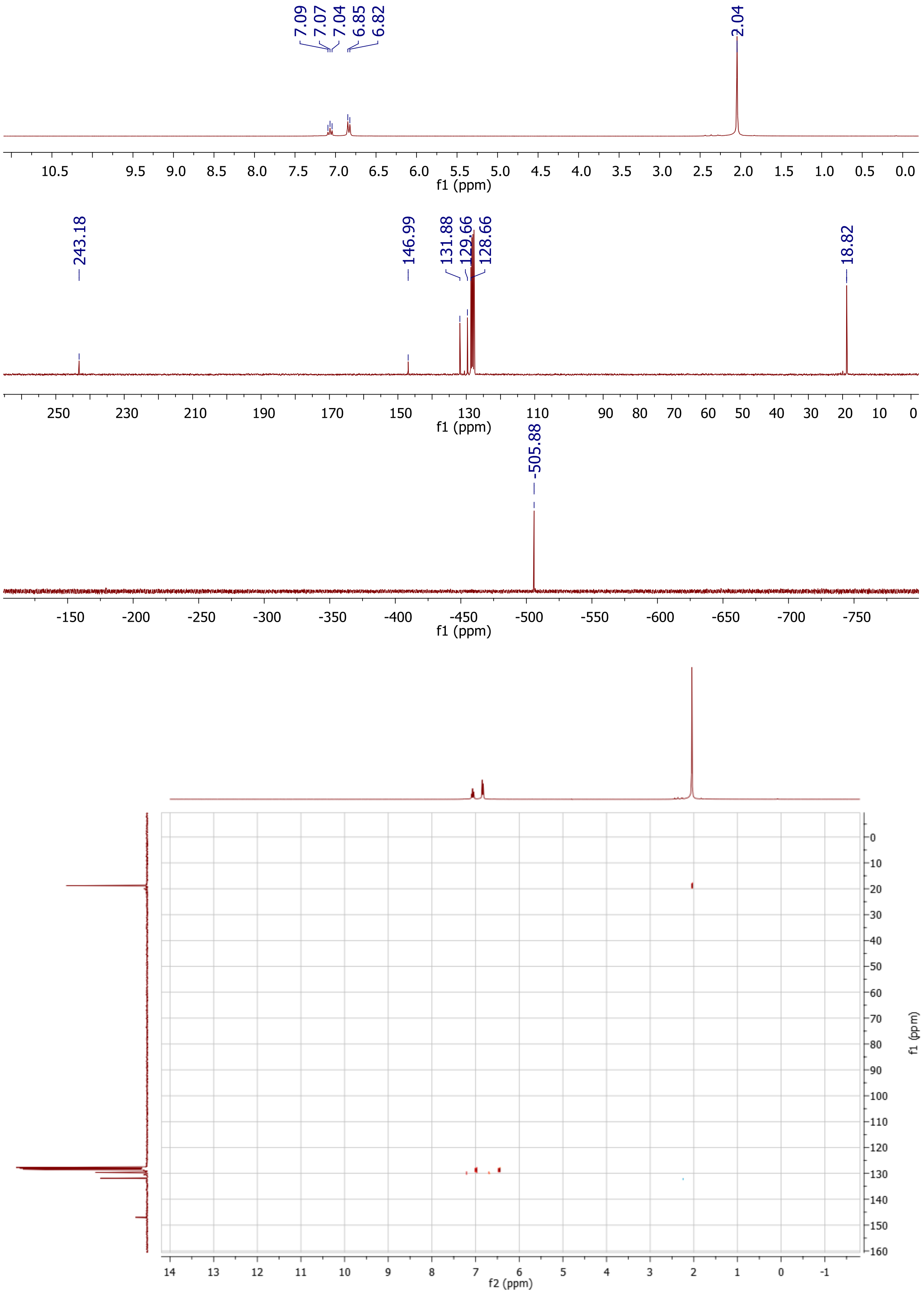

Steady-State Photolysis (SSP) / UV-Vis Spectroscopy

Figure S3: Comparison of the UV-Vis absorption spectra of compounds **1a,b** and **2–5** (samples: 0.6 mM solutions in acetonitrile).

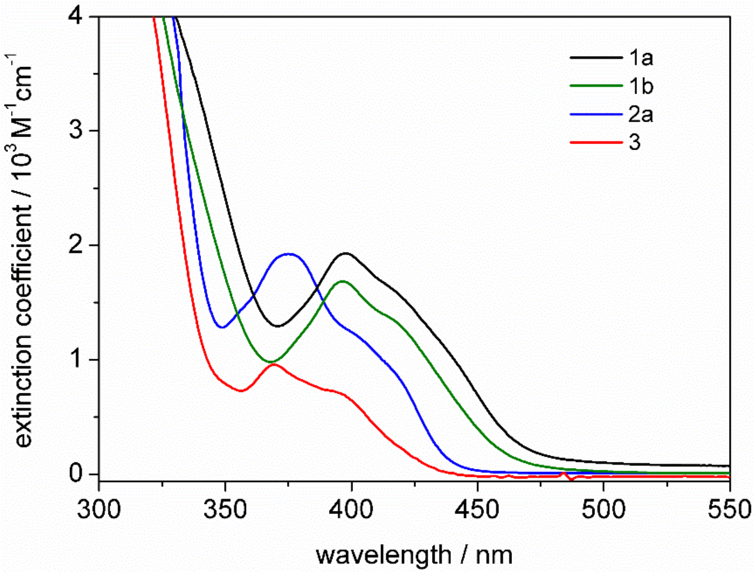

CIDNP Spectroscopy

Figure S4: ^{1}H NMR and CIDNP spectra of **1b** (10 mM solution in acetonitrile-d_3) recorded in presence of butyl acrylate **BA** (50 mM).

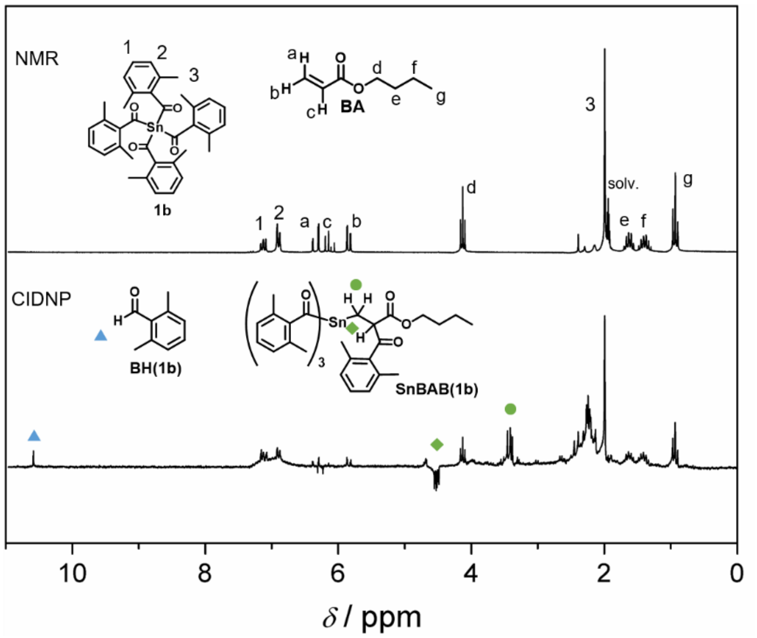

X-ray Crystallography

All crystals suitable for single crystal X-ray diffractometry were removed from a vial and immediately covered with a layer of silicone oil. A single crystal was selected, mounted on a glass rod on a copper pin, and placed in the cold N_2 stream provided by an Oxford Cryosystems cryostream. XRD data collection was performed for all compounds on a Bruker APEX II diffractometer with use of an Incoatec microfocus sealed tube of Mo Kα radiation (λ= 0.71073 Å) and a CCD area detector. Empirical absorption corrections were applied using SADABS or TWINABS.[6] The structures were solved with use of the intrinsic phasing option in SHELXT and refined by the full-matrix least-squares procedures in SHELXL.[7] The space group assignments and structural solutions were evaluated using PLATON.[8] Non-hydrogen atoms were refined anisotropically. Hydrogen atoms were located in calculated positions corresponding to standard bond lengths and angles. CCDC 1824373 and 1824374 contain the supplementary crystallographic data for all compounds. These data can be obtained free of charge from The Cambridge Crystallographic Data Centre *via* www.ccdc.cam.ac.uk/data_ request/cif. Table S1 contains crystallographic data and details of measurements and refinement for compounds **1a**,**b**.

Table S1: Crystallographic data and details of measurements for compounds **1a**,**b** Mo Kα (λ=0.71073Å). $R_1 = \Sigma / |F_o| - |F_c| / |\Sigma| F_d$; $wR2 = [\Sigma_w(F_o^2 - F_2^2)^2 / \Sigma_w(F_o^2)^2]^{1/2}$

Compound	**1a**	**1b**
Formula	$C_{40}H_{44}O_4Sn$	$C_{36}H_{36}O_4Sn$
Fw (g mol^{-1})	707.44	651.34
a (Å)	10.8064(4)	15.062(3)
b (Å)	12.0573(4)	11.477(2)
c (Å)	14.7267(6)	17.674(3)
α (°)	112.379(2)	90
β (°)	96.797(2)	90
γ (°)	94.499(2)	90
V (Å^3)	1745.83(11)	3055.2(10)
Z	2	4
Crystal size (mm)	0.15 × 0.13 × 0.12	0.16 × 0.13 × 0.09
Crystal habit	Block, yellow	Block, orange
Crystal system	Triclinic	Orthorhombic
Space group	*P*-1	$Pca2_1$
d_{calc} (mg/m^3)	1.346	1.416
μ (mm^{-1})	0.77	0.87
T (K)	100(2)	100(2)
2*θ* range (°)	2.6–27.7	2.3–33.1
F(000)	732	1336
R_{int}	0.107	0.084
independent reflns	6143	11687
No. of params	418	378
R1, wR2 (all data)	R1 = 0.0337<br>wR2 = 0.0744	R1 = 0.0390<br>wR2 = 0.0588
R1, wR2 (>2*σ*)	R1 = 0.0240<br>wR2 = 0.0516	R1 = 0.0297<br>wR2 = 0.0714

Photo-DSC

Table S2: Time to peak maximum t_{max}, maximum rate of photopolymerization $R_{P.max}$ and final double bond conversion DBC acquired from photo-DSC.

	PI [w%]	t_{max} [s]	$R_{P,max}$ [mol L^{-1} s^{-1}]	DBC [%]
**1a**	0.30	4.5	0.31	59 ± 1
**2a**	0.30	5.1	0.25	57 ± 1

Computational Section

All computational studies were executed on a computing cluster with blade architecture using the Gaussian09 software package.[9] Geometry optimizations were performed in the gas phase with B3LYP as hydride density functional[10] together with the LANL2DZdp-ECP for Sn[11] as well as 6-31+G(d,p) split-valence basis set for H, C and O.[12] Minimum structures were characterized by harmonic frequency calculations, yielding none imaginary frequency. Vertical excitations were calculated via time-dependent DFT (TD-DFT) at the LANL2DZdp-ECP(Sn),def2-TZVP(H,C,O) level of theory[13,14] and solvent effects were considered by applying the polarized continuum model (PCM).[15] Chloroform ($CHCl_3$) was used as a solvent with a dielectric constant of ε = 4.7113. All structures and the simulated UV-Vis spectra were plotted using the Gabedit software package.[16]

Table S3: Experimental and PCM($CHCl_3$) TD-DFT CAM-B3LYP/LANL2DZdp-ECP(Sn),def2-TZVP(H,C,O)//B3LYP/LANL2DZdp-ECP(Sn),6-31+G(d)(H,C,O) calculated Wavelength Absorption Maxima λ [nm], Extinction Coefficients ϵ [dm^2•mol^{-1}] and Oscillator Strengths f for **1a**,**b** as well as the corresponding trimethylstannane derivatives.

	$\lambda_{max,}$exp (ϵ)	Tetra $\lambda_{max,}$calc (f)	Mono $\lambda_{max,}$calc (f)	Absorption Edge	Assignment
**1a**	398 (1776)	416, 409, 389 (0.0221, 0.0051, 0.0137)	414 (0.0038)	480	n/σ-π*(CO/Aryl)
**1b**	396 (1735)	417, 409, 389 (0.0167, 0.0053, 0.0131)	415 (0.0036)	480	n/σ-π*(CO/Aryl)

Optimized cartesian coordinates and zero point vibrational energy corrected absolute energy in Hartree of **1a** (calculated with B3LYP/LANL2DZdp-ECP(Sn),6-31+G(d)(H,C,O)).

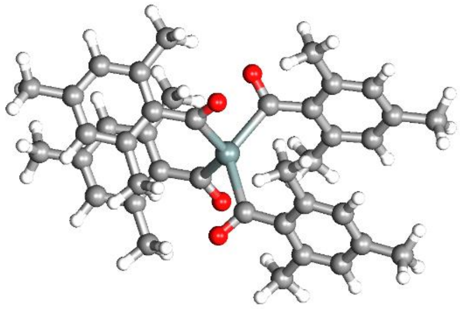

E = -1854.267064 a.u.

Sn	0.004788	0.000882	-0.002314
C	1.048907	-1.569791	1.231538
O	0.288015	-2.458006	1.587541
O	0.280764	2.460842	-1.592415
C	1.043562	1.573741	-1.237921
O	-0.284579	2.401092	1.678188
C	-1.042735	1.527086	1.283823
O	-0.284994	-2.400141	-1.682300
C	-1.043299	-1.527262	-1.285715
C	3.207094	-2.819545	1.019220
C	2.525476	-1.649753	1.437345
C	4.598763	-2.856536	1.144127
H	5.128930	-3.743851	0.803329
C	5.326272	-1.799947	1.700310
C	4.624677	-0.670237	2.131371
H	5.170484	0.155628	2.583198
C	3.237735	-0.567303	2.002069
C	2.488971	-4.015598	0.430343
H	3.208932	-4.697531	-0.033219
H	1.751500	-3.728439	-0.326217
H	1.945231	-4.568746	1.203311
C	2.537351	0.664647	2.531549
H	1.745941	0.412189	3.246112
H	2.069864	1.269303	1.745277
H	3.249879	1.316517	3.046406
C	6.825053	-1.892432	1.864204
H	7.090109	-2.349427	2.827305
H	7.294138	-0.903074	1.835238
H	7.277118	-2.507743	1.078931
C	2.519833	1.654659	-1.443841
C	3.232084	0.573082	-2.011302
C	3.200694	2.825765	-1.029483
C	2.530390	-0.655423	-2.547238
H	3.242687	-1.306293	-3.063668
H	1.740345	-0.398784	-3.261880
H	2.060744	-1.262665	-1.764239
C	6.825735	1.869820	-1.815154
H	7.304461	1.392813	-0.949234
H	7.181216	2.904778	-1.855145
H	7.186435	1.350453	-2.709723
C	2.482051	4.024986	-0.447763
H	1.744336	3.741316	0.309876
H	1.938558	4.574098	-1.223758
H	3.201584	4.709335	0.012881
C	-2.521551	1.598076	1.476877
C	-3.201029	2.781327	1.096136
C	-4.594721	2.809787	1.204314
H	-5.122476	3.710887	0.897906
C	-5.326591	1.728502	1.703439
C	-4.625976	0.588444	2.109450
H	-5.173828	-0.252193	2.530558
C	-3.237612	0.494427	1.994707
C	-2.478345	4.003518	0.569944
H	-1.941404	4.520410	1.372124
H	-3.194278	4.705979	0.131529
H	-1.734470	3.750422	-0.192446
C	-6.833676	1.782712	1.788527
H	-7.206851	1.227535	2.656059
H	-7.295765	1.338359	0.896396
H	-7.195002	2.813773	1.862580

C	-2.538569	-0.749871	2.496419
H	-1.755555	-0.515551	3.226250
H	-2.061280	-1.329957	1.697682
H	-3.254387	-1.418603	2.984356
C	-2.522547	-1.600304	-1.474645
C	-3.241426	-0.497753	-1.990907
C	-4.629992	-0.593519	-2.101605
H	-5.180085	0.246245	-2.521534
C	-5.328040	-1.734250	-1.693052
C	-4.593417	-2.814448	-1.195635
H	-5.119195	-3.716066	-0.887352
C	-3.199446	-2.784239	-1.091501
C	-2.545337	0.747176	-2.495211
H	-2.067291	1.328786	-1.698022
H	-3.263232	1.414337	-2.982251
H	-1.763505	0.513427	-3.226478
C	-6.835306	-1.790275	-1.773652
H	-7.195634	-2.821792	-1.846180
H	-7.211717	-1.235899	-2.640298
H	-7.295251	-1.346069	-0.880334
C	-2.473703	-4.005343	-0.567011
H	-1.727463	-3.751020	0.192639
H	-1.939038	-4.522224	-1.370735
H	-3.187387	-4.708277	-0.125684
C	4.592445	2.863998	-1.157304
H	5.121533	3.755345	-0.825647
C	5.320569	1.805184	-1.707403
C	4.618176	0.677340	-2.143533
H	5.162901	-0.144807	-2.603324

Optimized cartesian coordinates and zero point vibrational energy corrected absolute energy in Hartree of **1b** (calculated with B3LYP/LANL2DZdp-ECP(Sn),6-31+G(d)(H,C,O)).

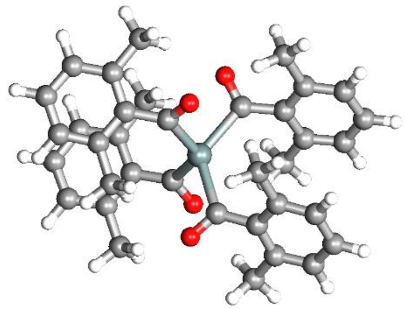

E = -1697.105806 a.u.

C	4.633268	0.630465	-2.100668	H	6.393759	-1.810497	1.721691
C	3.242043	0.537775	-1.998287	C	2.536890	0.683916	2.545037
C	2.530703	1.624968	-1.440875	C	-1.048651	1.547858	1.260731
C	3.203360	2.793537	-1.004892	C	-2.530744	1.624636	1.441060
C	4.598821	2.827337	-1.103243	C	-3.203737	2.793146	1.005422
C	5.310760	1.759479	-1.645551	C	-4.599181	2.826658	1.104106
C	1.048573	1.547896	-1.260874	C	-5.310785	1.758559	1.646378
O	0.291534	2.421334	-1.656143	C	-4.632969	0.629578	2.101100
C	2.537050	-0.683479	-2.545830	C	-3.241748	0.537184	1.998393
C	2.471827	3.989680	-0.433112	C	-2.472632	3.989538	0.433589
H	6.393853	1.810518	-1.720579	H	-6.393870	1.809361	1.721671
Sn	0.000013	0.000171	-0.000027	C	-2.536418	-0.684117	2.545401
C	-1.048525	-1.547579	-1.260895	O	0.291583	-2.421614	1.655066
C	-2.530631	-1.624689	-1.441012	O	-0.291754	2.421576	1.655670
C	-3.242004	-0.537353	-1.998114	O	-0.291421	-2.420931	-1.656233
C	-4.633228	-0.630038	-2.100549	H	5.129285	-3.711291	0.758066
C	-5.310699	-1.759213	-1.645801	H	5.185892	0.193944	2.544421
C	-4.598734	-2.827225	-1.103826	H	3.182640	-4.676385	-0.037880
C	-3.203279	-2.793417	-1.005424	H	1.724816	-3.704351	-0.314035
C	-2.537085	0.684117	-2.545288	H	1.938899	-4.538044	1.217272
H	-6.393789	-1.810257	-1.720858	H	1.759747	0.419407	3.270953
C	-2.471783	-3.989707	-0.433892	H	2.050913	1.287689	1.769155
C	1.048613	-1.547918	1.260325	H	3.250628	1.340539	3.052048
C	2.530663	-1.624912	1.440711	H	3.250867	-1.340151	-3.052666
C	3.203447	-2.793628	1.005294	H	1.760285	-0.418651	-3.272032
C	4.598882	-2.827452	1.104114	H	2.050574	-1.287276	-1.770268
C	5.310691	-1.759461	1.646305	H	1.724730	3.703975	0.314657
C	4.633089	-0.630270	2.100840	H	1.938107	4.537403	-1.216899
C	3.241904	-0.537570	1.998012	H	3.182103	4.676512	0.037920
C	2.472202	-3.989934	0.433464	H	-5.129781	3.710321	0.757907

H	-5.185614	-0.194711	2.544745
H	-1.939078	4.537478	1.217333
H	-3.183177	4.676099	-0.037432
H	-1.725464	3.704078	-0.314201
H	-1.759238	-0.419413	3.271203
H	-2.050413	-1.287815	1.769468
H	-3.249936	-1.340872	3.052550
H	-5.186139	0.194177	-2.543998
H	-5.129064	-3.711056	-0.757643
H	-2.051217	1.288034	-1.769444
H	-3.250852	1.340596	-3.052443
H	-1.759869	0.419588	-3.271111
H	-1.724671	-3.704146	0.313911
H	-1.938094	-4.537304	-1.217786
H	-3.182088	-4.676590	0.037016
H	5.129170	3.711042	-0.756767
H	5.186175	-0.193611	-2.544384

Optimized cartesian coordinates and zero point vibrational energy corrected absolute energy in Hartree of mesitoyltrimethylstannane (calculated with B3LYP/LANL2DZdp-ECP(Sn),6-31+G(d)(H,C,O)).

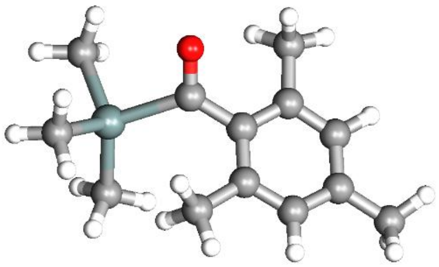

E = -585.731313 a.u.

C	1.329949	-3.539780	0.741244
C	1.124650	-3.231738	-0.623331
C	1.796659	-3.977328	-1.624560
C	2.615289	-5.040823	-1.232591
C	2.824069	-5.367253	0.110280
C	2.185996	-4.590075	1.081230
C	0.182495	-2.137352	-1.030248
O	0.474243	-1.344151	-1.920408
C	1.663699	-3.673985	-3.102023
C	3.701288	-6.533366	0.500003
C	0.700580	-2.729184	1.856486
Sn	-1.927876	-1.878463	-0.353924
C	-2.050191	-0.083159	0.830698
C	-2.960199	-1.588504	-2.218177
C	-2.783316	-3.558523	0.706400
H	-3.093918	0.228724	0.945122
H	-1.625318	-0.235527	1.828026
H	-1.501313	0.726837	0.339677
H	-3.806132	-3.737347	0.357691
H	-2.198850	-4.467505	0.530032
H	-2.817847	-3.374382	1.785117
H	-3.922547	-1.089140	-2.062934
H	-2.343703	-0.968623	-2.876092
H	-3.145589	-2.545919	-2.716480
H	3.116210	-5.624428	-2.002710
H	2.362333	-4.804204	2.133709
H	-0.370039	-2.941987	1.968452
H	1.172464	-2.971001	2.814125
H	0.809147	-1.650875	1.699495
H	4.163244	-6.377796	1.480761
H	3.119691	-7.463400	0.558283
H	4.500298	-6.696401	-0.231135
H	2.093584	-4.487621	-3.694997
H	0.621029	-3.541385	-3.408505
H	2.179612	-2.745206	-3.363288

Optimized cartesian coordinates and zero point vibrational energy corrected absolute energy in Hartree of 2,6-dimethylbenzoyltrimethylstannane (calculated with B3LYP/LANL2DZdp-ECP(Sn),6-31+G(d)(H,C,O)).

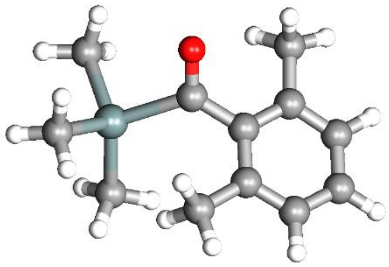

E = -546.441003 a.u.

C	1.374843	-3.487835	0.780919
C	1.164042	-3.197027	-0.586454
C	1.815430	-3.953829	-1.591862
C	2.632685	-5.020730	-1.200450
C	2.839985	-5.316350	0.144748
C	2.225013	-4.542989	1.126653
C	0.220214	-2.100292	-0.994378
O	0.532087	-1.278699	-1.849771
C	1.658951	-3.657501	-3.068493
H	3.487144	-6.142438	0.427670
C	0.754288	-2.656910	1.885770
Sn	-1.910607	-1.899591	-0.364452
C	-2.098198	-0.103127	0.809349
C	-2.913046	-1.644378	-2.250362
C	-2.729451	-3.599178	0.690893
H	-3.152426	0.170295	0.925470
H	-1.664485	-0.233886	1.805952
H	-1.581005	0.723610	0.311934
H	-3.759060	-3.780203	0.364123
H	-2.142825	-4.501152	0.488150
H	-2.739088	-3.430501	1.772646
H	-3.900699	-1.191034	-2.114454
H	-2.311657	-0.991397	-2.890187
H	-3.042174	-2.605551	-2.758950
H	3.120792	-5.618904	-1.966129
H	2.406872	-4.754789	2.177635
H	-0.320379	-2.850130	1.996740
H	1.219293	-2.895942	2.847453
H	0.880790	-1.582088	1.717481
H	2.084698	-4.470825	-3.664675
H	0.610713	-3.534042	-3.360642
H	2.165616	-2.726954	-3.341314

Simulation of UV-Vis spectra

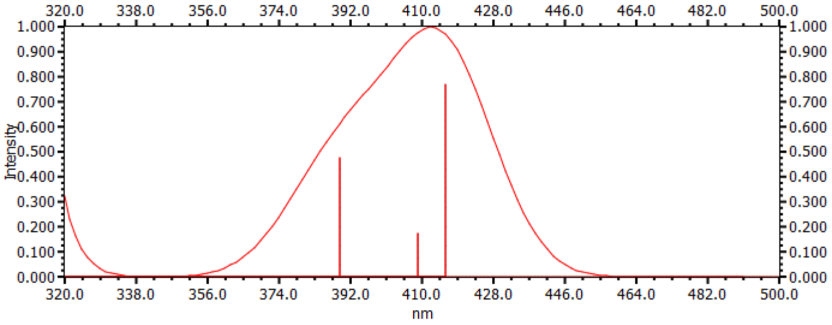

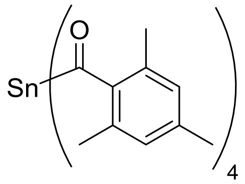

1a

Figure S5: Simulated UV-Vis spectrum of **1a** at the PCM($CHCl_3$) TD-DFT CAM-B3LYP/LANL2DZdp-ECP(Sn),def2-TZVP(H,C,O)//B3LYP/LANL2DZdp-ECP(Sn),6-31+G(d)(H,C,O) level of theory.

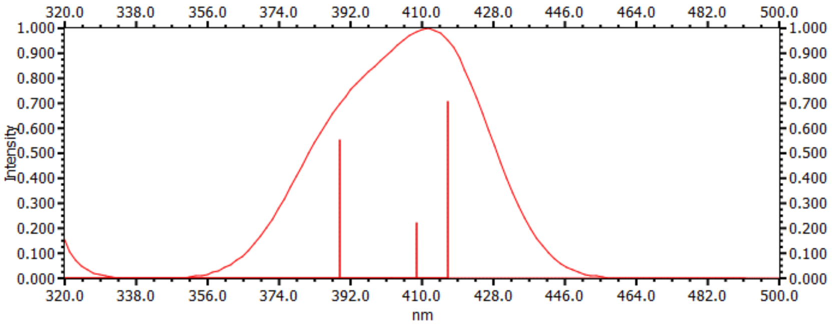

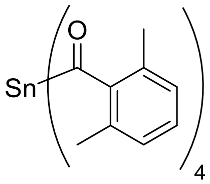

1b

Figure S6: Simulated UV-Vis spectrum of **1b** at the PCM($CHCl_3$) TD-DFT CAM-B3LYP/LANL2DZdp-ECP(Sn),def2-TZVP(H,C,O)//B3LYP/LANL2DZdp-ECP(Sn),6-31+G(d)(H,C,O) level of theory.

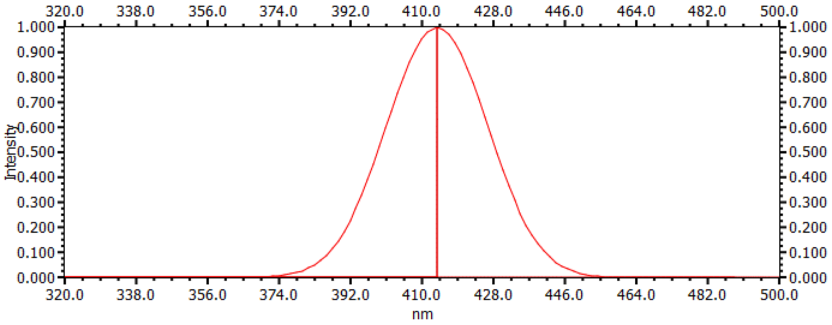

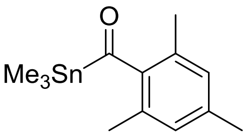

Figure S7: Simulated UV-Vis spectrum of mesitoyltrimethylstannane at the PCM($CHCl_3$) TD-DFT CAM-B3LYP/LANL2DZdp-ECP(Sn),def2-TZVP(H,C,O)//B3LYP/LANL2DZdp-ECP(Sn),6-31+G(d)(H,C,O) level of theory.

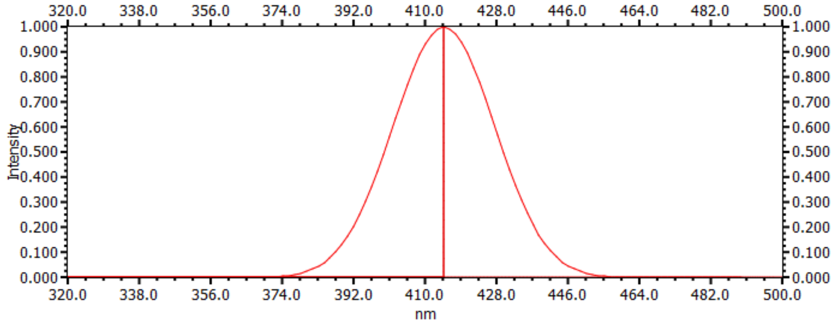

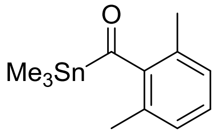

Figure S8: Simulated UV-Vis spectrum of 2,6-dimethylbenzoyltrimethylstannane at the PCM($CHCl_3$) TD-DFT CAM-B3LYP/LANL2DZdp-ECP(Sn),def2-TZVP(H,C,O)//B3LYP/LANL2DZdp-ECP(Sn),6-31+G(d)(H,C,O) level of theory.

Calculated UV-Vis spectrum of **1a** for the first ten singlet excitations at the PCM($CHCl_3$) TD-DFT CAM-B3LYP/LANL2DZdp-ECP(Sn),def2-TZVP(H,C,O)//B3LYP /LANL2DZdp-ECP(Sn),6-31+G(d)(H,C,O) level of theory.

```
 Excited State   1:      Singlet-A      2.9792 eV  416.17 nm  f=0.0221  <S**2>=0.000
     149 -> 163         0.15217
     150 -> 162        -0.15891
     158 -> 164        -0.30266
     159 -> 162         0.28674
     160 -> 161         0.46652
 This state for optimization and/or second-order correction.
 Total Energy, E(TD-HF/TD-KS) =  -1854.57798038
 Copying the excited state density for this state as the 1-particle RhoCI density.

 Excited State   2:      Singlet-A      3.0304 eV  409.14 nm  f=0.0051  <S**2>=0.000
     149 -> 164        -0.16043
     150 -> 161        -0.17832
     158 -> 163         0.31363
     159 -> 161         0.34640
     160 -> 162         0.40471

 Excited State   3:      Singlet-A      3.0884 eV  401.45 nm  f=0.0000  <S**2>=0.000
     149 -> 162         0.17046
     150 -> 163        -0.15826
     152 -> 161        -0.11829
     158 -> 161         0.42035
     159 -> 163         0.26820
     160 -> 164        -0.35889

 Excited State   4:      Singlet-A      3.1849 eV  389.29 nm  f=0.0137  <S**2>=0.000
     149 -> 161         0.17940
     150 -> 164         0.17941
     152 -> 162        -0.12075
     158 -> 162         0.37024
     159 -> 164        -0.27753
     160 -> 163         0.37282

 Excited State   5:      Singlet-A      4.3130 eV  287.47 nm  f=0.0836  <S**2>=0.000
     151 -> 164         0.16330
     152 -> 163         0.11688
     155 -> 162         0.21939
     157 -> 163        -0.12108
     159 -> 161         0.50162
     160 -> 162        -0.27106
     160 -> 165         0.13418

 Excited State   6:      Singlet-A      4.3590 eV  284.43 nm  f=0.2560  <S**2>=0.000
     150 -> 162         0.20724
     152 -> 164        -0.13000
     158 -> 164         0.25117
     158 -> 166         0.10052
```

159 -> 162 -0.27018
160 -> 161 0.49577

Excited State 7: Singlet-A 4.4640 eV 277.74 nm f=0.0006 <S**2>=0.000
155 -> 164 -0.10389
158 -> 161 0.49196
160 -> 164 0.43354
160 -> 166 0.13594

Excited State 8: Singlet-A 4.5246 eV 274.03 nm f=0.0362 <S**2>=0.000
150 -> 164 0.18470
151 -> 161 0.31155
152 -> 162 -0.16223
154 -> 161 0.13180
155 -> 163 -0.29058
157 -> 162 0.21814
158 -> 162 -0.24529
159 -> 164 0.26050
160 -> 163 0.10070

Excited State 9: Singlet-A 4.5421 eV 272.97 nm f=0.7916 <S**2>=0.000
150 -> 162 0.19718
151 -> 163 -0.22590
152 -> 164 -0.12836
154 -> 163 -0.13978
155 -> 161 0.36482
157 -> 164 0.22230
159 -> 162 0.37424

Excited State 10: Singlet-A 4.5573 eV 272.06 nm f=0.0405 <S**2>=0.000
150 -> 161 0.31028
151 -> 164 0.14626
152 -> 163 0.14332
154 -> 164 0.11005
155 -> 162 0.19308
157 -> 163 -0.23895
158 -> 163 -0.17776
160 -> 162 0.42776

Calculated UV-Vis spectrum of **1b** for the first ten singlet excitations at the PCM($CHCl_3$) TD-DFT CAM-B3LYP/LANL2DZdp-ECP(Sn),def2-TZVP(H,C,O)//B3LYP /LANL2DZdp-ECP(Sn),6-31+G(d)(H,C,O) level of theory.

Excited State 1: Singlet-A 2.9766 eV 416.53 nm f=0.0167 <S**2>=0.000
133 -> 147 -0.15218
135 -> 146 -0.12699
142 -> 146 0.29847
143 -> 148 0.31282
144 -> 145 0.47070
This state for optimization and/or second-order correction.
Total Energy, E(TD-HF/TD-KS) = -1697.35035259
Copying the excited state density for this state as the 1-particle RhoCI density.

Excited State 2: Singlet-A 3.0328 eV 408.82 nm f=0.0053 <S**2>=0.000
133 -> 148 -0.16107
135 -> 145 -0.14257
142 -> 145 0.36486
143 -> 147 0.32389
144 -> 146 0.40248

Excited State 3: Singlet-A 3.0810 eV 402.42 nm f=0.0000 <S**2>=0.000
133 -> 146 -0.16876
135 -> 147 -0.12776
136 -> 145 -0.10331
142 -> 147 0.28157
143 -> 145 0.43432
144 -> 148 0.36056

Excited State 4: Singlet-A 3.1848 eV 389.30 nm f=0.0131 <S**2>=0.000
133 -> 145 -0.17950
135 -> 148 -0.14776
136 -> 146 -0.10923
142 -> 148 0.29714
143 -> 146 0.38102
144 -> 147 0.37560

Excited State 5: Singlet-A 4.3729 eV 283.53 nm f=0.0714 <S**2>=0.000
134 -> 148 0.13109
136 -> 147 0.11484
137 -> 146 -0.18412
142 -> 145 0.49475
144 -> 146 -0.34515
144 -> 149 0.15374

Excited State 6: Singlet-A 4.3743 eV 283.44 nm f=0.1489 <S**2>=0.000
135 -> 146 0.15387
136 -> 148 0.11294
142 -> 146 -0.30348
142 -> 149 0.10591
143 -> 148 -0.27628

143 -> 150 -0.10477
144 -> 145 0.48903

Excited State 7: Singlet-A 4.4637 eV 277.76 nm f=0.0000 <S**2>=0.000
143 -> 145 0.48961
144 -> 148 -0.44677
144 -> 150 -0.14172

Excited State 8: Singlet-A 4.6144 eV 268.69 nm f=0.1089 <S**2>=0.000
134 -> 148 0.14167
135 -> 145 0.24794
136 -> 147 0.15902
137 -> 146 -0.18341
138 -> 145 0.24196
139 -> 148 0.17689
140 -> 146 -0.18940
141 -> 147 -0.25376
143 -> 147 -0.17691
144 -> 146 0.30591

Excited State 9: Singlet-A 4.6183 eV 268.46 nm f=0.0083 <S**2>=0.000
134 -> 145 -0.21871
135 -> 148 -0.11670
136 -> 146 -0.13431
137 -> 147 0.22455
142 -> 148 -0.22998
143 -> 146 -0.36516
144 -> 147 0.37499

Excited State 10: Singlet-A 4.6320 eV 267.67 nm f=0.0004 <S**2>=0.000
137 -> 147 -0.10259
138 -> 148 0.21003
139 -> 145 0.34949
140 -> 147 -0.25308
141 -> 146 -0.31303
143 -> 146 -0.14155
144 -> 147 0.21866

Calculated UV-Vis spectrum of mesitoyltrimethylstannane for the first ten singlet excitations at the PCM($CHCl_3$) TD-DFT CAM-B3LYP/LANL2DZdp-ECP(Sn),def2-TZVP(H,C,O)//B3LYP/LANL2DZdp-ECP(Sn),6-31+G(d)(H,C,O) level of theory.

Excited State 1: Singlet-A 2.9951 eV 413.96 nm f=0.0038 <S**2>=0.000
54 -> 56 -0.15006
55 -> 56 0.64122
55 -> 61 -0.17376
This state for optimization and/or second-order correction.
Total Energy, E(TD-HF/TD-KS) = -585.797317134
Copying the excited state density for this state as the 1-particle RhoCI density.

Excited State 2: Singlet-A 4.7124 eV 263.10 nm f=0.1648 <S**2>=0.000
53 -> 56 0.17342
53 -> 60 0.13455
54 -> 56 0.61418
54 -> 60 -0.11129
55 -> 56 0.13783

Excited State 3: Singlet-A 4.7892 eV 258.88 nm f=0.1621 <S**2>=0.000
53 -> 56 0.61130
54 -> 56 -0.19031
54 -> 60 -0.15503
55 -> 56 -0.14859

Excited State 4: Singlet-A 5.6502 eV 219.43 nm f=0.0698 <S**2>=0.000
53 -> 56 0.14752
55 -> 56 0.10758
55 -> 57 -0.16175
55 -> 59 -0.14359
55 -> 60 0.49151
55 -> 61 0.37647

Excited State 5: Singlet-A 5.7374 eV 216.10 nm f=0.0456 <S**2>=0.000
54 -> 56 -0.15284
55 -> 56 0.11682
55 -> 57 -0.15724
55 -> 58 -0.16289
55 -> 59 0.25628
55 -> 60 -0.30845
55 -> 61 0.44823

Excited State 6: Singlet-A 5.9872 eV 207.08 nm f=0.1050 <S**2>=0.000
53 -> 56 0.21339
53 -> 59 0.12837
53 -> 60 -0.29527
53 -> 61 -0.15637
54 -> 56 0.11005
54 -> 58 0.11393
54 -> 59 -0.20940
54 -> 60 0.40588

```
     54 -> 61        -0.15607
     55 -> 60        -0.14955

 Excited State   7:      Singlet-A      6.1258 eV  202.40 nm  f=0.1924  <S**2>=0.000
     53 -> 58         0.11598
     53 -> 59        -0.21005
     53 -> 60         0.40823
     53 -> 61        -0.15313
     54 -> 59        -0.14525
     54 -> 60         0.32739
     54 -> 61         0.24401

 Excited State   8:      Singlet-A      6.1886 eV  200.34 nm  f=0.0713  <S**2>=0.000
     52 -> 56         0.65575
     52 -> 61        -0.14262

 Excited State   9:      Singlet-A      6.2582 eV  198.11 nm  f=0.0201  <S**2>=0.000
     52 -> 56         0.10169
     55 -> 57         0.50369
     55 -> 58        -0.16136
     55 -> 59         0.31756
     55 -> 60         0.21176
     55 -> 68         0.11160

 Excited State  10:      Singlet-A      6.3440 eV  195.44 nm  f=0.0148  <S**2>=0.000
     51 -> 56         0.64603
     51 -> 61        -0.11985
     55 -> 57         0.14873
```

Calculated UV-Vis spectrum of 2,6-dimethylbenzoyltrimethylstannane for the first ten singlet excitations at the PCM($CHCl_3$) TD-DFT CAM-B3LYP/LANL2DZdp-ECP(Sn),def2-TZVP(H,C,O)//B3LYP/LANL2DZdp-ECP(Sn),6-31+G(d)(H,C,O) level of theory.

Excited State 1: Singlet-A 2.9884 eV 414.88 nm f=0.0036 <S**2>=0.000
49 -> 52 -0.13153
51 -> 52 0.64927
51 -> 57 -0.18551
This state for optimization and/or second-order correction.
Total Energy, E(TD-HF/TD-KS) = -546.490896916
Copying the excited state density for this state as the 1-particle RhoCI density.

Excited State 2: Singlet-A 4.7633 eV 260.29 nm f=0.0608 <S**2>=0.000
49 -> 52 -0.12531
49 -> 56 0.20281
50 -> 52 0.63076

Excited State 3: Singlet-A 4.9058 eV 252.73 nm f=0.1922 <S**2>=0.000
49 -> 52 0.64284
50 -> 52 0.12090
51 -> 52 0.15942

Excited State 4: Singlet-A 5.5850 eV 222.00 nm f=0.0424 <S**2>=0.000
49 -> 52 -0.10924
51 -> 52 0.16185
51 -> 53 -0.18395
51 -> 56 0.35001
51 -> 57 0.52139

Excited State 5: Singlet-A 5.7539 eV 215.48 nm f=0.0393 <S**2>=0.000
50 -> 52 -0.15106
51 -> 53 0.12301
51 -> 54 0.10718
51 -> 55 -0.32357
51 -> 56 0.48685
51 -> 57 -0.28132

Excited State 6: Singlet-A 6.0868 eV 203.69 nm f=0.0865 <S**2>=0.000
48 -> 52 0.10437
49 -> 55 -0.23532
49 -> 56 0.45345
50 -> 52 -0.23385
50 -> 56 0.12255
50 -> 57 0.30716
51 -> 56 -0.11044

Excited State 7: Singlet-A 6.1749 eV 200.79 nm f=0.0967 <S**2>=0.000
47 -> 52 -0.14103
48 -> 52 0.30893

49 -> 56 -0.14266
49 -> 57 -0.23227
50 -> 55 -0.24184
50 -> 56 0.44504

Excited State 8: Singlet-A 6.1839 eV 200.50 nm f=0.1793 <S**2>=0.000
48 -> 52 0.57429
48 -> 57 -0.13199
49 -> 57 0.12179
50 -> 55 0.14472
50 -> 56 -0.28463

Excited State 9: Singlet-A 6.3043 eV 196.67 nm f=0.0194 <S**2>=0.000
47 -> 52 -0.30625
51 -> 53 0.39256
51 -> 54 -0.17981
51 -> 55 0.36425
51 -> 56 0.19118
51 -> 63 0.10982

Excited State 10: Singlet-A 6.3263 eV 195.98 nm f=0.0196 <S**2>=0.000
47 -> 52 0.58341
47 -> 57 -0.11813
48 -> 52 0.15416
51 -> 53 0.26380
51 -> 55 0.13952

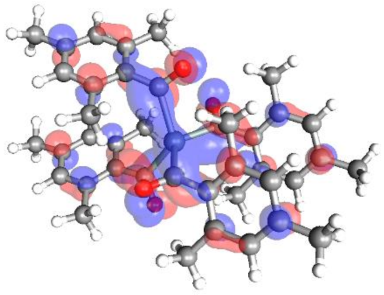

-0.62 eV

LUMO+1

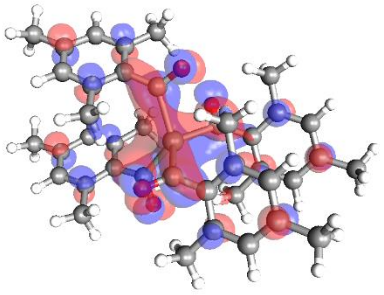

-0.91 eV

LUMO

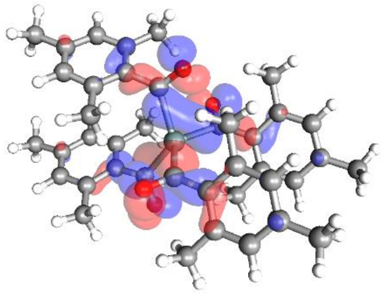

-7.46 eV

HOMO

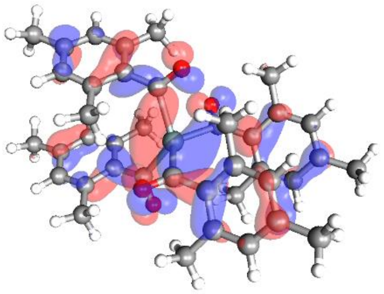

-7.61 eV

HOMO-1

Figure S9: HOMO-1, HOMO, LUMO and LUMO+1 together with orbital energies of **1a** at the PCM($CHCl_3$) TD-DFT CAM-B3LYP/LANL2DZdp-ECP(Sn),def2-TZVP(H,C,O)//B3LYP/LANL2DZdp-ECP(Sn),6-31+G(d)(H,C,O) level of theory.

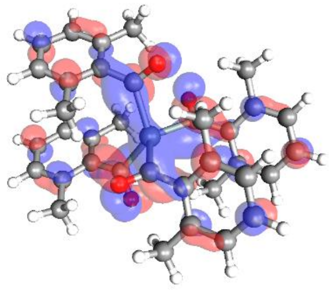

-0.70 eV

LUMO+1

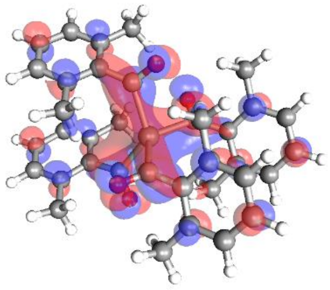

-1.02 eV

LUMO

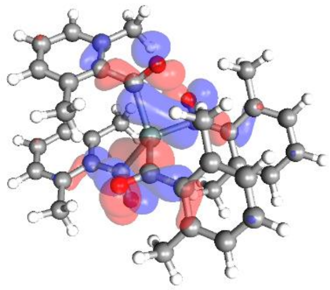

-7.59 eV

HOMO

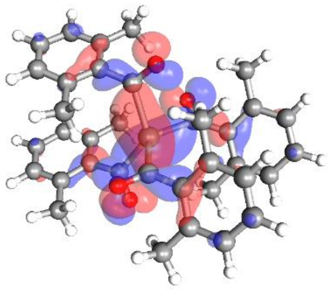

-7.76 eV

HOMO-1

Figure S10: HOMO-1, HOMO, LUMO and LUMO+1 together with orbital energies of **1b** at the PCM($CHCl_3$) TD-DFT CAM-B3LYP/LANL2DZdp-ECP(Sn),def2-TZVP(H,C,O)//B3LYP/LANL2DZdp-ECP(Sn),6-31+G(d)(H,C,O) level of theory.

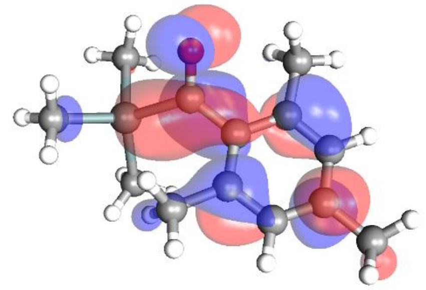

-0.42 eV

LUMO

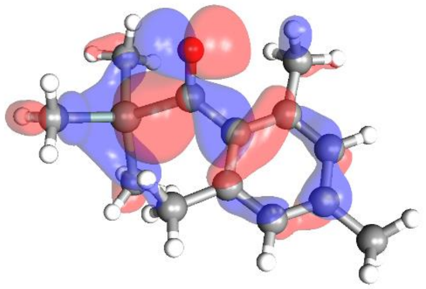

-7.54 eV

HOMO

Figure S11: HOMO and LUMO together with orbital energies of mesitoyltrimethylstannane at the PCM($CHCl_3$) TD-DFT CAM-B3LYP/LANL2DZdp-ECP(Sn),def2-TZVP(H,C,O)//B3LYP/LANL2DZdp-ECP(Sn),6-31+G(d)(H,C,O) level of theory.

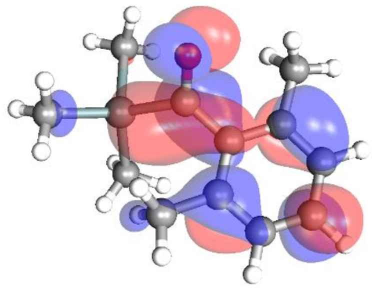

-0.46 eV

LUMO

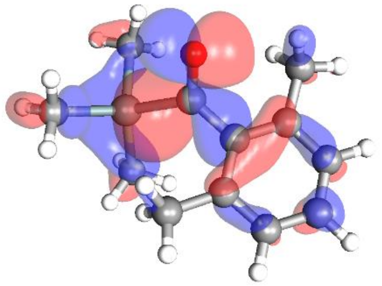

-7.60 eV

HOMO

Figure S12: HOMO and LUMO together with orbital energies of 2,6-dimethylbenzoyltrimethylstannane at the PCM($CHCl_3$) TD-DFT CAM-B3LYP/LANL2DZdp-ECP(Sn),def2-TZVP(H,C,O)//B3LYP /LANL2DZdp-ECP(Sn),6-31+G(d)(H,C,O) level of theory.

Tabel S4: NBO analysis of mesitoyltrimethylstannane at the PCM($CHCl_3$) TD-DFT CAM-B3LYP/LANL2DZdp-ECP(Sn),def2-TZVP(H,C,O)//B3LYP/LANL2DZdp-ECP(Sn),6-31+G(d)(H,C,O) level of theory.

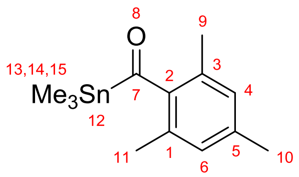

			%	%s	%p	%d	%f	H.O.
C1-C2	□	C1	49.18	34.02	65.83	0.14	0.01	$sp^{1.94}$
		C2	50.82	34.84	65.10	0.06	0.01	$sp^{1.87}$
C1-C2	□	C1	45.70	0.00	99.92	0.04	0.03	sp
		C2	54.30	0.05	99.91	0.02	0.02	$sp^{99.99}d^{0.28}f^{0.37}$
C1-C6	□	C1	50.52	34.86	65.01	0.12	0.01	$sp^{1.86}$
		C6	49.48	35.95	63.90	0.14	0.01	$sp^{1.78}$
C1-C11	□	C1	50.19	31.08	68.88	0.03	0.01	$sp^{2.22}$
		C11	49.81	29.46	70.33	0.19	0.01	$sp^{2.39}d^{0.01}$
C2-C3	□	C2	50.98	34.95	64.99	0.06	0.01	$sp^{1.86}$
		C3	49.02	33.76	66.09	0.15	0.01	$sp^{1.96}$
C2-C7	□	C2	53.02	30.09	69.80	0.09	0.02	$sp^{2.32}$
		C7	46.98	37.59	62.34	0.06	0.01	$sp^{1.66}$
C3-C4	□	C3	50.39	34.62	65.25	0.12	0.01	$sp^{1.88}$
		C4	49.61	36.03	63.82	0.14	0.01	$sp^{1.77}$
C3-C4	□	C3	46.54	0.00	99.93	0.04	0.03	sp
		C4	53.46	0.00	99.94	0.03	0.03	sp
C3-C9	□	C3	50.59	31.59	68.38	0.03	0.01	$sp^{2.16}$
		C9	49.41	29.37	70.43	0.19	0.01	$sp^{2.40}$
C4-C5	□	C4	49.65	35.81	64.01	0.16	0.01	$sp^{1.79}$
		C5	50.35	34.29	65.59	0.11	0.01	$sp^{1.91}$
C5-C6	□	C5	50.29	34.24	65.64	0.11	0.01	$sp^{1.92}$
		C6	49.71	35.81	64.02	0.16	0.01	$sp^{1.79}$
C5-C6	□	C5	46.64	0.01	99.94	0.04	0.02	sp
		C6	53.36	0.00	99.94	0.03	0.03	sp
C5-C10	□	C5	50.43	31.43	68.53	0.04	0.01	$sp^{2.18}$
		C10	49.57	29.73	70.06	0.01	0.01	$sp^{2.36}d^{0.01}$
C7-O8	□	C7	33.36	30.90	68.99	0.09	0.01	$sp^{2.23}$
		O8	66.64	42.77	56.62	0.59	0.02	$sp^{1.32}d^{0.01}$
C7-O8	□	C7	31.34	0.76	99.09	0.14	0.01	$sp^{99.99}d^{0.18}f^{0.01}$
		O8	68.66	0.56	99.05	0.37	0.02	$sp^{99.99}d^{0.66}f^{0.03}$
C7-Sn12	□	C7	74.91	31.31	68.65	0.04	0.01	$sp^{2.19}$
		Sn12	25.09	21.93	77.87	0.19	-	$sp^{3.55}d^{0.01}$
Sn12-C13	□	Sn12	24.56	26.11	73.79	0.10	-	$sp^{2.83}$
		C13	75.44	26.02	73.94	0.03	0.00	$sp^{2.84}$
Sn12-C14	□	Sn12	24.45	25.95	73.96	0.08	-	$sp^{2.85}$
		C14	75.55	26.14	73.83	0.03	0.00	$sp^{2.82}$
Sn12-C15	□	Sn12	24.33	26.01	73.85	0.14	-	$sp^{2.84}d^{0.01}$
		C15	75.67	25.94	74.03	0.03	0.00	$sp^{2.85}$
LP1	-	O8	-	56.50	43.39	0.10	0.00	$sp^{0.77}$
LP2	-	O8	-	0.06	99.69	0.24	0.01	$sp^{99.99}d^{4.03}f^{0.22}$

Tabel S5: NBO analysis of 2,6-dimethylbenzoyltrimethylstannane at the PCM($CHCl_3$) TD-DFT CAM-B3LYP/LANL2DZdp-ECP(Sn),def2-TZVP(H,C,O)//B3LYP/LANL2DZdp-ECP(Sn),6-31+G(d)(H,C,O) level of theory.

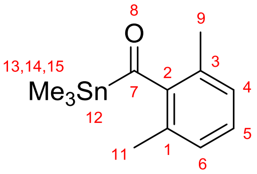

			%	%s	%p	%d	%f	H.O.
C1-C2	□	C1	49.15	33.96	65.89	0.14	0.01	$sp^{1.94}$
		C2	50.85	34.97	64.97	0.06	0.01	$sp^{1.86}$
C1-C6	□	C1	50.69	34.80	65.08	0.11	0.01	$sp^{1.87}$
		C6	49.31	35.67	64.17	0.14	0.01	$sp^{1.80}$
C1-C6	□	C1	47.81	0.00	99.93	0.04	0.03	sp
		C6	52.19	0.00	99.94	0.03	0.03	sp
C1-C11	□	C1	50.24	31.19	68.77	0.03	0.01	$sp^{2.20}$
		C11	49.76	29.45	70.35	0.19	0.01	$sp^{2.39}d^{0.01}$
C2-C3	□	C2	50.98	35.10	64.83	0.06	0.01	$sp^{1.85}$
		C3	49.02	33.73	66.12	0.14	0.01	$sp^{1.96}$
C2-C3	□	C2	53.88	0.01	99.96	0.02	0.02	sp
		C3	46.12	0.00	99.92	0.05	0.03	sp
C2-C7	□	C2	53.02	29.85	70.04	0.10	0.01	$sp^{2.35}$
		C7	46.98	37.40	62.53	0.06	0.01	$sp^{1.67}$
C3-C4	□	C3	50.57	34.56	65.32	0.11	0.01	$sp^{1.89}$
		C4	49.43	35.75	64.10	0.14	0.01	$sp^{1.79}$
C3-C9	□	C3	50.61	31.67	68.29	0.03	0.01	$sp^{2.16}$
		C9	49.39	29.34	70.46	0.19	0.01	$sp^{2.40}$
C4-C5	□	C4	50.13	35.70	64.14	0.14	0.01	$sp^{1.80}$
		C5	49.87	35.69	64.18	0.12	0.01	$sp^{1.80}$
C4-C5	□	C4	51.78	0.00	99.94	0.03	0.03	sp
		C5	48.22	0.00	99.95	0.03	0.02	sp
C5-C6	□	C5	49.82	35.63	64.23	0.12	0.01	$sp^{1.80}$
		C6	50.18	35.69	64.16	0.14	0.01	$sp^{1.80}$
C7-O8	□	C7	33.32	30.75	69.16	0.09	0.01	$sp^{2.25}$
		O8	66.68	42.65	56.75	0.59	0.01	$sp^{1.33}d^{0.01}$
C7-O8	□	C7	31.52	0.94	98.91	0.14	0.01	$sp^{99.99}d^{0.14}$
		O8	68.48	0.75	98.86	0.37	0.02	$sp^{99.99}d^{0.50}$
C7-Sn12	□	C7	75.05	31.45	68.50	0.04	0.01	$sp^{2.18}$
		Sn12	24.95	21.80	78.03	0.17	-	$sp^{3.58}d^{0.01}$
Sn12-C13	□	Sn12	24.62	26.13	73.77	0.10	-	$sp^{2.82}$
		C13	75.38	25.96	74.00	0.03	0.00	$sp^{2.85}$
Sn12-C14	□	Sn12	24.51	25.97	73.95	0.08	-	$sp^{2.85}$
		C14	75.49	26.08	73.89	0.03	0.00	$sp^{2.83}$
Sn12-C15	□	Sn12	24.41	26.10	73.78	0.12	-	$sp^{2.83}$
		C15	75.59	25.89	74.08	0.03	0.00	$sp^{2.86}$
LP1	-	O8	-	56.45	43.45	0.10	0.00	$sp^{0.77}$
LP2	-	O8	-	0.05	99.70	0.24	0.01	$sp^{99.99}d^{5.01}f^{0.28}$

References

[1] A. B. Pangborn, M. A. Giardello, R. H. Grubbs, R. K. Rosen, F. J. Timmers, *Organometallics* **1996**, *15*, 1518.

[2] a) L. R. Subramanian, G. Siegemund (Eds.) *Houben-Weyl Methods of Organic Chemistry Supplement: Organo-Fluorine Compounds Fluorinating Agents and Their Application in Organic Synthesis*. Introduction of Fluorine with Alkali Metal Fluorides, Including Ammonium Fluoride and Tetraalkylammonium Fluorides (Including Special Methods of Fluorinations, e.g., Phase Transfer Catalysis, Activation by Crown Ethers, Reagents Bound to Polymers), Thieme, Stuttgart, **1998**; b) U. G. H. Buerger, *Angew. Chem. Int. Ed.* **1968**, *7*, 212.

[3] J. Radebner, A. Eibel, M. Leypold, C. Gorsche, L. Schuh, R. Fischer, A. Torvisco, D. Neshchadin, R. Geier, N. Moszner et al., *Angew. Chem. Int. Ed.* **2017**, *56*, 3103.

[4] K. S. Anseth, C. M. Wang, C. N. Bowman, *Macromolecules* **1994**, *27*, 650.

[5] a) G. Ullrich, B. Ganster, U. Salz, N. Moszner, R. Liska, *J. Polym. Sci. A Polym. Chem.* **2006**, *44*, 1686; b) M. K. Mishra, Y. Yagci, M. K. H. o. r. v. p. Mishra, *Handbook of vinyl polymers*. *Radical polymerization, process, and technology / editors, Munmaya Mishra and Yusuf Yagci*, Taylor & Francis, Boca Raton, FL, **2008**.

[6] a) Bruker, *APEX2 and SAINT*. *version 5.03*. Bruker AXS Inc.:, Madison, Wisconsin, USA, **2012**; b) R. H. Blessing, *Acta Cryst.* **1995**, *51*, 33.

[7] a) G. M. Sheldrick, *Acta Crystallogr., Sect. A: Found. Crystallogr.* **1990**, *A46*, 467; b) G. M. Sheldrick, *Acta Crystallogr., Sect. A: Found. Crystallogr.* **2008**, *64*, 112; c) G. M. Sheldrick, *Acta Crystallogr., Sect. A: Found. Adv.* **2015**, *71*, 3.

[8] a) A. L. Spek, *J. Appl. Crystallogr.* **2003**, *36*, 7; b) A. L. Spek, *Acta Crystallogr., Sect. D: Biol. Crystallogr.* **2009**, *65*, 148.

[9] M. J. Frisch, G. W. Trucks, H. B. Schlegel, G. E. Scuseria, M. A. Robb, J. R. Cheeseman, G. Scalmani, V. Barone, B. Mennucci, G. A. Petersson, H. Nakatsuji, M. Caricato, X. Li, H. P. Hratchian, A. F. Izmaylov, J. Bloino, G. Zheng, J. L. Sonnenberg, M. Hada, M. Ehara, K. Toyota, R. Fukuda, J. Hasegawa, M. Ishida, T. Nakajima, Y. Honda, O. Kitao, H. Nakai, T. Vreven, Jr. J. A. Montgomery, J. E. Peralta, F. Ogliaro, M. Bearpark, J. J. Heyd, E. Brothers, K. N. Kudin, N. Staroverov, T. V. Keith, R. Kobayashi, J. Normand, K. Raghavachari, A. Rendell, J. C. Burant, S. S. Iyengar, J. Tomasi, M. Cossi, N. Rega, J. M. Millam, M. Klene, J. E. Knox, J. B. Cross, V. Bakken, C. Adamo, J. Jaramillo, R. Gomperts, R. E. Stratmann, O. Yazyev, A. J. Austin, R. Cammi, C. Pomelli, J. W. Ochterski, R. L. Martin, K. Morokuma, V. G. Zakrzewski, G. A. Voth, P. Salvador, J. J. Dannenberg, S. Dapprich, A. D. Daniels, O. Farkas, J. B. Foresman, J. V. Ortiz, J. Cioslowski, D. J. Fox, Gaussian 09, Revision D.01; Gaussian, Inc.: Wallingford, CT, **2013**.

[10] (a) A. D. Becke, *J. Chem. Phys.* **1993**, *98*, 1372. b) C. Lee, W. Yang, R. Parr, *Phys. Rev. B: Condens. Matter Mater. Phys.* **1988**, *37*, 785.

[11] P. J. Hay, W. R. Wadt, *J. Chem. Phys*. **1985**, *82*, 270. b) P. J. Hay, W. R. Wadt, *J. Chem. Phys.* **1985**, *82*, 284. c) P. J. Hay, W. R. Wadt, *J. Chem. Phys*. **1985**, *82*, 299. (d) C. E. Check, T. O. Faust, J. M. Bailey, B. J. Wright, T. M. Gilbert, L. S. Sunderlin, *J. Phys. Chem. A* **2001**, *105*, 8111.

[12] a) P. C. Hariharan, J. A. Pople, *Theor. Chim. Acta* **1973**, *28*, 213. (b) W. J. Hehre, R. Ditchfield, J. A. Pople, *J. Chem. Phys.* **1972**, *56*, 2257. c) R. Ditchfield, W. J. Hehre, J. A. Pople, *J. Chem. Phys.* **1971**, *54*, 724.
[13] T. Yanai, D. Tew, N. Handy*, Chem. Phys. Lett*. **2004**, *393*, 51.
[14] F. Weigend, R. Ahlrichs, *Phys. Chem. Chem. Phys.* **2005**, *7*, 3297.
[15] J. Tomasi, B. Mennucci, R. Cammi, *Chem. Rev.* **2005**, *105*, 2999.
[16] A. R. Allouche, *J. Comput. Chem.* **2011**, *32*, 174.
